# Optimizing mouse models for mRNA vaccines: addressing dose translation challenges

**DOI:** 10.1038/s41598-026-47820-z

**Published:** 2026-05-05

**Authors:** Zi Wei Chang, Yong Jie Tan, Yiyu Liao, Yun Shan Goh, Syed Ridwan Kabir, Yuqian Wang, Pei Xiang Hor, Chiew Yee Loh, Yuling Huang, Bei Wang, Siti Nazihah Mohd Salleh, Eve Zi Xian Ngoh, COVID-19 Cohort Study Group, Angeline Rouers, Anthony Torres-Ruesta, Nathan Wong, Alice Soh Meoy Ong, Adeline Chiew Yen Chua, Samantha Nguee, Vanessa Neo, Isaac Kai Jie Kam, Ajayanandan Yadunandan, Sooriya Kannan Selvam, Jarvis Goh, Ng Kah Ying, Sim Xin Yi, Wong Wei Lun, Anna Xinyi Loo, Liang Hui Loo, NCID Study Group, Jocelyn Jin Yu, Zheng Kuang Soh, Yi Qing Chin, Jonathan Jordon Lim, Juwinda Ongko, Eshele Anak Libau, Celine Theo, Mohammed Ridzwan Bin Abdullah, Shiau Hui Diong, He Ping Yeo, COVID-19 Clinicians’ Group, Surinder Pada, Louisa Jin Sun, Desmond Luan Seng Ong, Jyoti Somani, Eng Sing Lee, Cheng-I Wang, David C. Lye, Barnaby E. Young, Matthew Zirui Tay, Lisa F. P. Ng, Keisuke Ejima, Laurent Renia

**Affiliations:** 1 A*STAR Infectious Diseases Labs (A*STAR IDL), Agency for Science, Technology and Research (A*STAR), Singapore, Singapore; 2 Lee Kong Chian School of Medicine, Nanyang Technological University, 11 Mandalay Road, Level 12, Singapore, 308232 Singapore; 3 School of Biological Sciences, Nanyang Technological University, Singapore, Singapore; 4 A*STAR Singapore Immunology Network (A*STAR SIgN), Agency for Science, Technology and Research (A*STAR), Singapore, Singapore; 5 National Centre for Infectious Diseases, Singapore, Singapore; 6 Department of Biochemistry, Yong Loo Lin School of Medicine, National University of Singapore, Singapore, Singapore; 7 Department of Infectious Diseases, Tan Tock Seng Hospital, Singapore, Singapore; 8 Yong Loo Lin School of Medicine, National University of Singapore, Singapore, Singapore; 9 Communicable Diseases Agency, Singapore, Singapore; 10 Ng Teng Fong General Hospital, Singapore, Singapore; 11 Infectious Diseases, Alexandra Hospital, Singapore, Singapore; 12 National University Polyclinic, Singapore, Singapore; 13 Division of Infectious Diseases, Department of Medicine, National University Hospital, National University Health System, Singapore, Singapore; 14 National Healthcare Group Polyclinics, Singapore, Singapore

**Keywords:** Mouse, Human, Dose translation, Antibodies, mRNA vaccine, BNT162b2, Diseases, Immunology

## Abstract

The development of mRNA vaccines has significantly advanced global health during the COVID-19 pandemic. However, translating effective vaccine doses from preclinical animal models to human clinical use remains poorly understood. In this study, we evaluated antibody responses and repertoire diversity across different BNT162b2 mRNA vaccine doses in mice, while comparing the results with human data through mathematical modeling. We observed that 1 μg BNT162b2 dose regimen induced a persistent antibody response and broad antibody repertoire for up to 16 weeks in mice. Conversely, 0.05 μg BNT162b2 dose regimen resulted in waning antibody responses and narrowed antibody repertoires over the same period, resembling responses observed in humans vaccinated with 30 μg BNT162b2 dose regimen. An empirical dose translation approach revealed a discrepancy in dose relevance between the 1 μg commonly used in the in vivo mice model and the 30 μg used for vaccination in human. Therefore, it is crucial to re-evaluate the appropriate mRNA vaccine dose for in vivo mice models to accurately reflect human responses.

**Supplementary Information:**

The online version contains supplementary material available at 10.1038/s41598-026-47820-z.

## Introduction

In response to the COVID-19 pandemic, multiple vaccine platforms, including mRNA^1,2^, viral vector^3^, inactivated virus^4^, and recombinant protein-based vaccines^5^, were rapidly developed and authorized for emergency use. By August 2024, 13.72 billion doses had been administered^6^. While mRNA vaccines were previously assessed against other infectious diseases^7^, their use significantly expanded after the COVID-19 vaccination roll out.

In preclinical models, including rodents^8–11^and non-human primates^10^, the COVID-19 mRNA vaccines demonstrated a broad effective dose range (0.2 to 100 μg). Within this broad range, dose-saturation effect has been observed beyond 1 µg of mRNA vaccine that in mouse model^12,13^. However, there is no clear or defined strategy for determining the optimal dose for subsequent human clinical trials. Furthermore, the pharmacokinetics difference in mRNA delivery between preclinical and clinical settings remains inadequately characterized. Usually, to convert a mouse dose to a human equivalent dose (HED), body surface area (BSA) normalization is the most widely accepted method^14^. This approach accounts for metabolic differences between species and avoids overestimating doses for use in humans. In mice, a 1–10 μg vaccine dose is usually selected since these have been shown in many studies to be immunogenic. Using BSA, a standard conversion factor is ~ 12.3 × (based on average mouse: human weight ratio of 0.02 kg:70 kg), translating a 10 μg mouse dose to a ~ 123 μg dose for humans^15^. Thus, doses approved for human use in later clinical trials ranged from 30 to 400 μg^2,16,17^.

While conventional dose conversion across species typically involves parameters such as body surface area (as mentioned above) and metabolic rate differences^14^, there are various host factors at play that are unexplored. mRNA vaccine delivery is complex, requiring transportation via lipid nanoparticles, release through endocytosis, subsequent translation into protein by ribosomes, and the ability to trigger an immune response for functionality^18^. Due to enhanced delivery systems, such as lipid nanoparticles, which improve cellular uptake^16^, mRNA vaccines may need lower-than-predicted doses to achieve the same effect. It has also been shown that these factors may be specific to the vaccine delivery system used for mRNA vaccine and should be considered during mRNA vaccine dose translation calculation. In fact, BNT162b2 and mRNA1273 mRNAs have been reported in most studies to persist in humans for up to 30 days post-vaccination^19,20^. However, recent reports have detected the SARS-CoV-2 spike protein in mice and humans after vaccination for extended periods, i.e. up to 4 years^21,22^. Even though the protein expression linearly correlates to the amount of mRNA in vivo^23^, studies in humans have shown an absence of correlation between the level of mRNA in the blood and the levels of spike-binding IgG or neutralizing antibodies^19^. Prior study has shown that mice immunized with a two-dose regimen of 1 μg BNT162b2 maintained an antibody response against the SARS-CoV-2 ancestral strain and its Variants of Concern (VOCs) for up to 36 weeks^24^. These findings contrast with observations in humans. Individuals vaccinated with a two-dose BNT162b2 regimen typically show a waning antibody response and a poor response to SARS-CoV-2 VOCs within three weeks to five months following primary vaccination^25,26^.

With the advancement and wider use of mRNA vaccines, understanding dose translation from preclinical to the clinical settings is essential, but remains poorly elucidated. Therefore, we utilized BNT162b2 vaccine humoral response data from the mouse in vivo model and computational modeling to investigate gaps in our knowledge of mRNA dose translation into humans.

## Results

### Long term and sustained antibody responses in mice immunized with 1 μg of BNT162b2

**Fig. 1:**
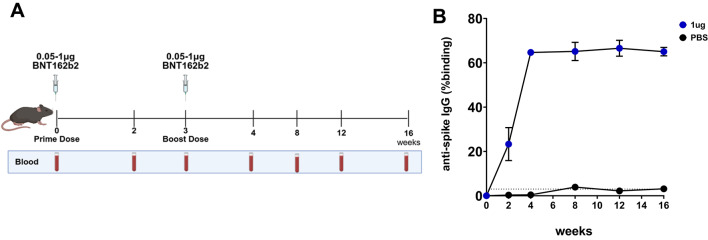
Humoral responses of mice immunized with 1 μg BNT162b2. (**A**) Schematic diagram of vaccination schedule in C57BL/6 mice. (**B**) Longitudinal IgG binding antibodies responses against ancestral strain full-length spike in mice after immunization with 2 doses of 1 μg of BNT162b2. Data are presented as median with interquartile range. The dotted line denotes the assays lower limit of detection.

Because a 2 dose regimen of 1 µg of mRNA was shown to induce an immune response in BNT162b2 immunized mice^10^, we assessed the antibody responses in mice following the 2 dose regimen (3 weeks apart) of 1 μg of BNT162b2 up to 16 weeks (Fig. 1A). Using this regimen, no waning of binding antibody levels was observed over the 16 weeks (Fig. 1B). This is starkly different from the antibody responses in humans, where after a 2 dose regimen antibody waning occurs 12 weeks post-vaccination^27^. We hypothesized that the immunization dose in mice may have been too high. Thus, we assessed lower doses of 0.05, 0.1, 0.2 and 0.5 μg.

**Fig. 2:**
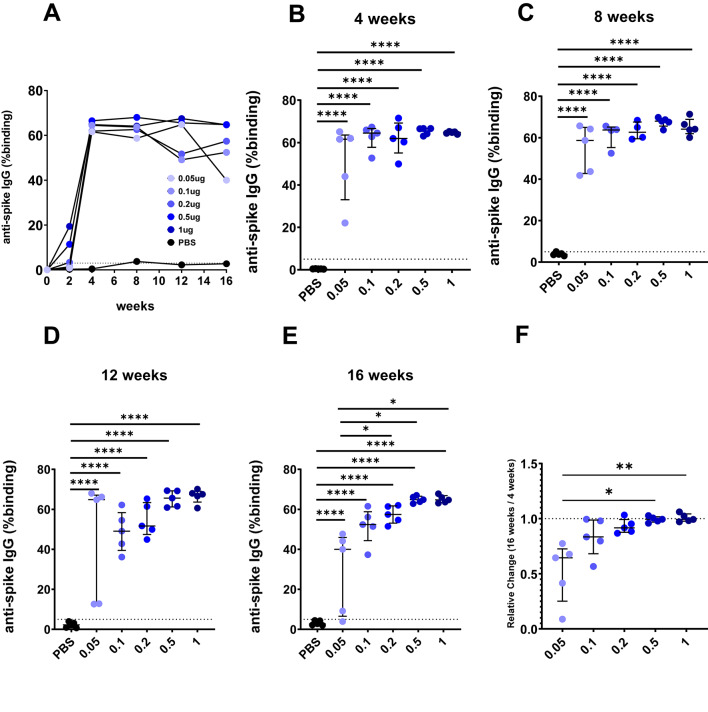
Comparison of humoral responses in mice immunized with various doses of BNT162b2 up to 16 weeks. (**A**) Longitudinal IgG binding antibodies responses against ancestral strain full-length spike in mice immunized with 2 doses of 0.05, 0.1, 0.2, 0.5 or 1 μg of BNT162b2 and control mice receiving PBS. Data are presented as median. Comparison of IgG responses against ancestral strain full-length spike protein in mice at 4 (**B**), 8 (**C**), 12 (**D**) and 16 (**E**) weeks. The dotted line in each panel denotes the assays lower limit of detection. (**F**) Relative change in IgG binding responses against ancestral strain full-length spike between 4 and 16 weeks. The dotted line denotes no relative change in IgG binding responses against ancestral strain full-length spike between 4 and 16 weeks. *, *p* < 0.05, **, *p* < 0.01, ****, *p* < 0.0001 (one-way ANOVA followed by Tukey’s multiple comparisons test). Data are presented as median with interquartile range.

Mice immunized with 2 dose regimens of BNT162b2 across all various doses have significant antibody responses against control mice received phosphate-buffered saline (PBS). Indeed, waning antibody responses were observed in mice immunized with 2 dose regimen of 0.05 μg BNT162b2 (Fig. 2A). Comparable antibody responses were observed across all evaluated doses at 4 (Fig. 2B), 8 (Fig. 2C) and 12 (Fig. 2D) weeks. However, at 16 weeks, binding antibody responses in 0.05 μg immunized mice were significantly lower compared to the 0.2 μg, 0.5 μg and 1 μg groups (Fig. 2E). Moreover, the relative change in the antibody responses between 4 and 16 weeks was significantly lower in 0.05 μg 2 dose regimen immunized mice compared to the 0.5 μg and 1 μg 2 dose regimen at 16 weeks groups (Fig. 2F). Taken together, antibody response persisted up to 16 weeks at higher doses, while waning was more prominent in 0.05 μg immunized mice at 16 weeks.

### Broader antibody repertoires against SARS-CoV-2 VOCs in mice immunized with 2 doses of 1 μg of BNT162b2

**Fig. 3:**
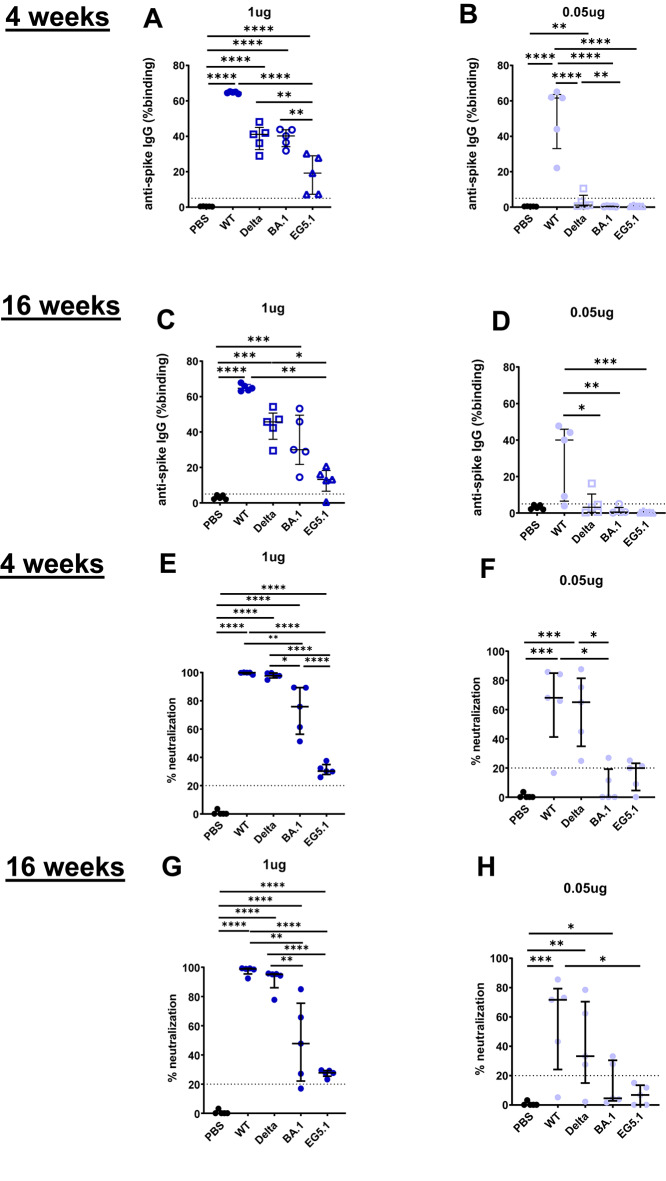
Comparison of cross-reactivity in mice immunized with 1 μg and 0.05 μg of BNT162b2 at 4 and 16 weeks. Comparison of IgG responses against ancestral, Delta, Omicron BA.1 and EG5.1 strain full-length spike in mice immunized with 2 doses of 1 μg (**A**) and 0.05 μg (**B**) of BNT162b2 measured at 4 weeks post first immunization. Comparison of IgG binding responses against ancestral, Delta, Omicron BA.1 and EG5.1 strain full-length spike in mice immunized with 2 doses of 1 μg (**C**) or 0.05 μg (**D**) of BNT162b2 at 16 weeks. Data are presented as median with interquartile range. Comparison of neutralizing antibodies responses against ancestral, Delta, Omicron BA.1 and EG5.1 strain full-length spike in mice immunized with 1 μg (**E**) and 0.05 μg (**F**) of BNT162b2 prime and boost at 4 weeks. Comparison of neutralizing antibodies responses against ancestral, Delta, Omicron BA.1 and EG5.1 strain full-length spike in mice immunized with 2 doses with 1 μg (**G**) or 0.05 μg (**H**) of BNT162b2 at 16 weeks. *, *p* < 0.05, **, *p* < 0.01, ***, *p* < 0.001, ****, *p* < 0.0001 (one-way ANOVA followed by Tukey’s multiple comparisons test). Data are presented as median with interquartile range. The dotted line in each panel denotes the assays lower limit of detection.

In addition to the strength of the vaccine-induced antibody responses, we also assessed the breadth of the antibody repertoire. At 4 weeks, we observed antibody responses, against the SARS-CoV-2 ancestral strain, and variants of concern (VOCs) including Delta, and Omicron subvariants BA.1 and EG.5.1. The antibody levels against the ancestral strain were significantly higher than those against the SARS-CoV-2 VOCs at 4 weeks (Fig. 3A) and this persisted until 16 weeks (Fig. 3C; Supplementary Fig. 1A). In contrast, we did not detect binding antibody responses against Delta, and Omicron subvariants BA.1 and EG.5.1 in mice immunized with 0.05 μg of BNT162b2 (Fig. 3B, D and Supplementary Fig. 1B), even at 4 weeks. Similarly, neutralizing antibody responses in mice immunized with 2 doses of 1 μg followed the same trend. Neutralizing antibody responses against VOCs were detected in mice immunized with 2 doses of 1 μg at 4 weeks (Fig. 3E) and this persisted until 16 weeks (Fig. 3G or Supplementary Fig. 1C). Comparatively, only neutralizing antibody responses against WT and Delta were observed in mice immunized with 2 doses of 0.05 μg (Fig. 3F), and these further waned at week 16 (Fig. 3H and Supplementary Fig. 1D). Our data demonstrates that higher doses elicit a broader antibody repertoire against SARS-CoV-2 VOCs, persisting for up to 16 weeks.

### Estimated antibodies trajectories in BNT162b2 vaccinated mice and humans

To understand dose translation from mice to human, we utilized mice humoral response data from this study and humoral response data from young adults in a previously published study^28^. Both human and mouse binding antibody responses were collected using the same SFB assay. The humoral response data were obtained from young adults (< 60 years) in Singapore who were vaccinated with the BNT162b2, based on longitudinal blood samples collected at baseline (the day of the first dose), 21 days later at the time of the second dose, and up to 180 days post-immunization. We fitted the developed mathematical model for mice and human antibody data.

**Fig. 4:**
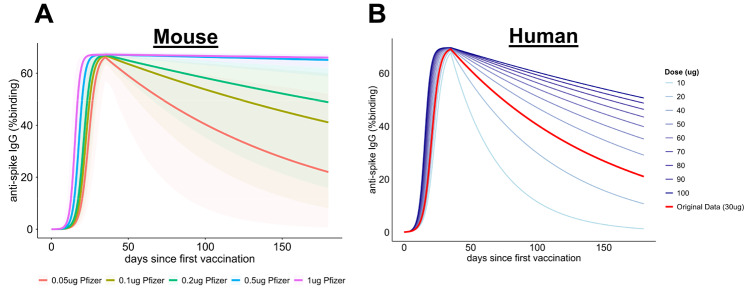
Comparison of antibody trajectories in mice immunized with different doses and scaled to humans. (**A**) Fitted trajectories of anti-spike IgG binding (% binding) in mice immunized with 2 doses of 0.05 μg, 0.1 μg, 0.2 μg, 0.5 μg, or 1 μg, modelled over 150 days since first vaccination. Shaded lighter region represent 95% confidence intervals of the fitted model. The red dashed line represents the original data: antibody trajectory in mice with 0.05 ug dose. (**B**) Human anti-spike IgG binding trajectories obtained via dose translation from the mouse data, with doses ranging from 10 to 100 μg plotted over 150 days since first vaccination. The red dashed line represents the original data: antibody trajectory in human with 30 ug dose.

In mice, higher doses led IgG levels to approach the maximum capacity $C$, more rapidly, resulting in an apparently visual peak (Fig. 4A). This pattern is explained by higher ${k}_{1}$, which causes the trajectory to reach the plateau more quickly within the fixed growth phase (Supplementary Fig. 2A). After the peak, the IgG level starts to decline in lower dose regimen groups (0.05–0.2 μg), while the IgG level was maintained in the 0.5 μg and 1 μg 2 dose regimen groups, explained by negative association between ${k}_{2}$ and dose (Supplementary Fig. 2B). Notably, when the dose is more than 0.5 μg dose regimen, the decay rate reaches close to zero. The fitting for each mouse is available in Supplementary Fig. 3 and estimated parameters are available in Supplementary Table 1. We fitted the model to human data as well (the red line in Fig. 4B), with individual fits detailed in Supplementary Fig. 4 and estimated parameters are available in Supplementary Table 2. Sensitivity testing showed that relaxing the transition time ($\tau$) did not meaningfully improve model fit for both mice and humans (Supplementary Table 3), we therefore retained the fixed-peak model to ensure ${k}_{1}$ and ${k}_{2}$ remained comparable across groups.The association between ${k}_{1}$ and dose and that between ${k}_{2}$ and dose were the best explained by the logarithmic model and power-law model, respectively (Supplementary Fig. 2). AIC and R^2^ for each model are available in Supplementary Table 4 and specific coefficients of these equations are provided in Supplementary Table 5.

Lastly, for the human data, the model parameters for different doses were extrapolated using the dose translation approach and the corresponding IgG trajectory was estimated (Fig. 4B). As we observed in the model fitting to the mice data, higher doses are associated with faster growth (higher ${k}_{1}$) and slower decay (lower ${k}_{2}$). Interestingly, even with extremely high dose of 100ug, we observed substantial waning, which is because ${k}_{2}$ saturates with high doses (Supplementary Fig. 2). This qualitative finding is consistent with the human trial with different doses, where waning was observed with the doses as high as 60 μg^29^. Nevertheless, the extrapolations beyond clinically tested ranges remain purely model-based and should be interpreted as theoretical estimates of high-dose kinetics. To mitigate the uncertainty brought by the translation factor, we performed sensitivity analysis using different translation factors (e.g. 10, 50, 200) (Supplementary Fig. 5). Across all dose levels, the trajectories for different translation factors closely overlap. Results showed that for the same human dose, the corresponding extrapolated parameters under different SF revealed limited difference in the parameters. This indicates that our qualitative finding—specifically the discrepancy in dose relevance between 1 μg in mice and 30 μg in humans—is independent of the specific translation factor chosen.

In addition to dose translation, we conducted pairwise statistical comparisons of antibody model parameters to identify which mouse dose (0.05, 0.1, 0.2, 0.5, or 1 μg) most closely approximates the human response at a 30 μg dose. Interestingly, we found that the antibody decay rate, $k2$, was statistically equivalent between 0.05 μg in mice and human response (Welch’s two-sample t-test: *p* = 0.627; Two One-Sided Tests (TOST): *p* = 8.38e-03) (Supplementary Fig. 7A). In contrast, k2, was statistically different between 1 μg in mice and human response ((Welch’s two-sample t-test: *p* = 1.02e-79; Two One-Sided Tests (TOST): p = 1)) (Supplementary Fig. 7B). Our study reveals that the antibody response trajectory of the 0.05 μg two-dose regimen in mice more closely resembles the human response, including its cross-reactivity against SARS-CoV-2 VOCs. However, it is important to note that this similarity is driven primarily by the decay rate $k2$; the initial growth rate $k1$ remained statistically higher in all mouse dose groups compared to humans (Supplementary Fig. 7A). This suggests that while waning dynamics can be matched, induction kinetics may differ across species.

## Discussion

Our study investigates the in vivo antibody response to the BNT162b2 mRNA vaccine, specifically focusing on the dose relevance between human and mouse models. Through a combination of in vivo experiments and mathematical modelling, we identified a notable discrepancy in antibody response at high doses of the mRNA vaccine when comparing mouse and human studies. Previous in vivo studies in mice with BNT162b2 primarily focused on understanding immune mechanisms for up to 4 to 6 weeks^30^. Our current data aligns with a prior study^24^, confirming that mice immunized with a two-dose regimen of 1 μg BNT162b2 maintained an antibody response against the SARS-CoV-2 ancestral strain and its Variants of Concern (VOCs) for up to 36 weeks. However, these findings contrast with observations in humans.

Individuals vaccinated with a two-dose BNT162b2 regimen typically show a waning antibody response and a poor response to SARS-CoV-2 VOCs within three weeks to five months following primary vaccination^25,26^. Importantly, our study and others have found that the antibody response in mice receiving a 1 μg two-dose mRNA vaccine reached a plateau, indicating that the immune response becomes saturated at higher doses^12,13^. While we have not explored the underlying mechanisms of this observation, increased antigen expression does leads to expand of memory B cell populations^31,32^. The expansion may have reach plateau in preclinical model but this remained to be confirmed. Moreover, this suggests that maximal mRNA vaccine response in mice occurs at high doses, which may not accurately reflect human immune response dynamics. Furthermore, the cross-reactivity against SARS-CoV-2 variants of concern (VOCs) in these mice does not match the human response to the two-dose BNT162b2 vaccination. This suggests that a lower mRNA dose in mice might better represent human immune response dynamics in particular antibody waning rate and antibody repertoires. However, our empirical dose-translation approach assumes that the mathematical relationships between antibody kinetic parameters and dose—logarithm and power-law observed in mice model—are fundamentally conserved in humans. This is a significant assumption, as human responses may be subject to different biological constraints. While our current model aligns qualitatively with clinical data up to 60 μg^29^, these cross-species projections remain theoretical at higher doses.

We note a particular limitation in our study in that it primarily focuses on dose relevance based on antibody response and does not consider cellular responses. Assessment of dose translation in the context of cellular responses remains a challenge, as monitoring cellular responses in vivo is difficult due to the inability to track the same mice longitudinally. Moreover, the dose dependency of T cell responses is not straightforward, as these data were limited or absent in individuals receiving 1–50 μg two-dose regimens of BNT162b2^30^. The objective of this study was to assess dose translation for the primary mRNA vaccination series. We did not evaluate the longevity of subsequent antibody responses following booster doses or with other types of RNA vaccine platforms.

As COVID-19 became endemic, most individuals who received the ancestral strain vaccine as their primary vaccination series would likely have either been infected or boosted with a variant of concern. These subsequent exposures may lead to immune imprinting and the back-boosting effect^33–35^. Moreover, this effect has been observed in in vivo studies where repeated exposures of later strains are required to overcome immune imprinting^36,37^. While we observed a similar humoral response trend between humans and mice vaccinated with low doses, it would be worthwhile to investigate whether a comparable immune imprinting pattern occurs in humans and mice receiving additional doses of variants of concern.

With the emergence of more mRNA vaccine platforms for infectious diseases and their potential application in other conditions, it is essential to establish their effectiveness using appropriate in vivo doses that accurately recapitulate human immunogenicity. We recommend assessing a wide range of doses to avoid dose saturation effect and include very low doses to precisely determine antibody longevity and waning in preclinical model. This facilitates bridging preclinical studies to predict human responses and real-world applications. Ultimately, deriving a “conversion factor” might be less about a single number and more about a predictive model that considers these interacting biological factors. This model is grounded in the Immunostimulation/Immunodynamic (IS/ID) mathematical modeling framework advocated by Rhodes et al.^38^, which employs immunodynamic models and allometric scaling to translate vaccine immunogenicity across species. This integrated approach enables researchers to forecast human immunogenicity more accurately based on mouse data, thus streamlining preclinical development.

## Methods

### Mice

Six to eight-week-old female C57BL/6 J mice, purchased from Invivos (Singapore), were used in this study. The mice were housed under specific pathogen-free conditions in the A*STAR Biological Resource Centre (BRC), Singapore. All experiments and procedures were performed in accordance with the Animal & Veterinary Service (AVS) and National Advisory Committee for Laboratory Animal Research (NACLAR) of Singapore, under the approval of the Institutional Animal Care and Use Committee (IACUC #211673).

### Immunization

BNT162b2 (Pfizer) encodes the ancestral strain of SARS-CoV-2 antigen^10^ and was obtained and approved for use by the Ministry of Health, Singapore. Mice were immunized with 50 µL of vaccine containing 0.05 to 1 µg of BNT162b2 per dose on days 0 and 21, as per previous studies^10,39–41^. Control mice received phosphate-buffered saline (PBS). Mouse sera were collected under anesthesia using isoflurane on days 0, 14, and 28, and monthly thereafter, for measurement of humoral response (Fig. 1A). At the end of the experiment, the mice will be euthanized using CO2 at a displacement rate of 30%. Schematic diagrams of the vaccination schedule in C57BL/6 J mice were created with BioRender.com.

### Spike protein flow cytometry-based assay (SFB assay) for antibody detection

To capture the full range of antibodies, including those that bind to different domains and conformational epitopes of the S protein, the SFB assay was adapted from a previously described method for antibody detection^42,43^. HEK 293 cells expressing the spike protein^42^ of the ancestral strain and variants of concern were seeded at 2 × 10^4^ cells/well in 96-well V-bottom plates (ThermoFisher Scientific, USA). Cells were incubated with mouse serum (diluted 1:10,800 in 10% FBS; HyClone, USA), followed by a secondary incubation with a double stain containing Alexa Fluor 647-conjugated anti-mouse IgG (1:600 dilution; ThermoFisher Scientific) and propidium iodide (PI; 1:2500 dilution; Sigma-Aldrich, Burlington, USA). Cells were acquired using a BD Biosciences Symphony A5 SE (New Jersey, USA) and analyzed with FlowJo software (Tree Star, BD Biosciences). Live cells (PI negative) were gated, and binding was determined by the percentage of GFP-positive, S protein-expressing cells bound by antibody, as indicated by Alexa Fluor 647- and FITC-positive events. Expression of the different spike proteins from the ancestral strain and variants of concern on the surface of the cell lines used in this study was confirmed by ACE-2-HuFc binding in a previous study^25^ and Supplementary Fig. 6. The relative change in the antibody responses between 4 and 16 weeks was calculated by dividing the antibody responses at 16 weeks by antibody responses at 4 weeks for each mouse. The value of 1 would indicate no waning while the value lower than 1 would indicate waning.

### Pseudovirus neutralization assay

The pseudotyped lentivirus neutralization assay was performed according to a previously described protocol with slight modifications^44^. Briefly, CHO-ACE2, a stable cell line expressing human ACE2, was a kind gift from Dr. Yee-Joo Tan (Department of Microbiology, National University of Singapore & Institute of Molecular and Cell Biology, A*STAR, Singapore)^45^ and was utilized for the assay. The CHO-ACE2 cells were seeded at 1.8 × 10^4^ cells per well in a 96‐well black microplate (Corning, USA) with DMEM without Geneticin and were allowed to settle overnight. Mouse plasma samples were diluted (1:400 dilutions) and were incubated with an equal volume of pseudovirus-expressing spike proteins of the respective SARS-CoV-2 strain (5 ng of p24 per well) at 37 °C for 1 h. The mixture was then added in duplicate to the pre‐seeded CHO‐ACE2 cells. The wells were topped up with DMEM after 1 h of incubation. After 48 h, cells were washed with PBS and lysed with 1X Passive Lysis Buffer (Promega) with gentle shaking at 125 rpm at 37 °C for 30 min. Luciferase activity was subsequently quantified using the Luciferase Assay System (Promega) on a GloMax Luminometer (Promega).

### Human study cohort for humoral response analysis

The humoral response data from young adults in this study were previously published in a study^28^. The study design and protocol were reviewed and approved by the National Healthcare Group (NHG) Domain Specific Review Board (DSRB) under study number 2012/00917. Participants gave written informed consent in line with the Declaration of Helsinki for Human Research. Blood samples were collected from healthy individuals who received 2 doses of the mRNA vaccine BNT162b2 on days 0, 21, and up to 180 days after the first dose. None of the participants had known or reported SARS-CoV-2 infection.

### Estimating the antibody response to mRNA vaccination in humans at different doses using mice data

Data on antibody response to vaccination in humans at different doses are useful for determining optimal dosing strategies. However, due to ethical and safety limitations, testing multiple doses directly in humans is challenging. To address this, we aimed to estimate the antibody response to different vaccine doses in humans using data from mice studies, in which various doses have been tested. 6- to 8-week-old mice were used in the corresponding preclinical vaccine immunogenicity study and are considered young adult in their immune system development^46^. Thus, the human participants aged ≥ 60 years were excluded from subsequent analyses to align with the age range used in the human and mouse studies. Indeed, immune responses to COVID-19 mRNA vaccination differ markedly in adults ≥ 60 years, who exhibit reduced magnitude, slower kinetics, and lower neutralizing and binding antibody titers compared with younger adults^28,47^.

We use a mathematical model that describes antibody kinetics and an empirical dose translation method to extrapolate mouse doses and dose–response relationships to humans^38^. There are three steps: (A) development of a mathematical model to describe temporal change in antibody level after vaccination and fitting the model to both mice and human data (i.e., estimating model parameters), (B) estimation of dose-parameter relationship in the mouse data, (C) extrapolation of human antibody responses at other doses by applying the relative parameter changes observed in mice. There are two important assumptions for this approach. First, the dose translation factor between mouse and humans for the mRNA is constant. For example, 0.01, 0.05, 0.1, 0.2, and 1 μg, for mice correspond to 1, 5, 10, 20, and 100 μg for human, with a translation factor of 100. Second, proportional change in the parameters due to different doses is consistent in mice and human (explained further in C. Extrapolation of antibody response to vaccination in human under different doses using empirical dose translation).

A) Process 1. Development of a mathematical model to describe temporal change in antibody level after vaccination and fitting the model to both mice and human data.

We developed a mathematical model to characterize the antibody dynamics post-vaccination. This model captures the initial sigmoid growth (i.e., it initially rises slowly, then rapidly approaches an exponential rate, and finally slows down, stabilizing at a carrying capacity) phase of IgG levels, followed by exponential decay after reaching a peak. The antibody level $A(t)$ (expressed as a percentage of IgG binding) at time $t$ (days after the first dose) is defined as:

$$A\left(t\right)=\left\{\begin{array}{c}\begin{array}{cc}\frac{C{A}_{0}{e}^{{k}_{1}t}}{C +{A}_{0}\left({e}^{{k}_{1}t}-1\right)}& \left(t\le \tau \right)\\ {A}_{peak}{e}^{{-k}_{2}t}& \left(t>\tau \right)\end{array}\end{array},\right.$$

where ${A}_{0}$ and ${A}_{peak}$ are the IgG level at day 0 (i.e., $A(0)$) and day $\tau$ (i.e., $\frac{C{A}_{0}{e}^{{k}_{1}\tau }}{C +{A}_{0}\left({e}^{{k}_{1}\tau }-1\right)}$), $C$ is the individual’s IgG capacity which is the maximum level of IgG level one can reach, ${k}_{1}$ and ${k}_{2}$ are the growth rate and the decay rate of IgG level, respectively. $\tau$ is the time of transition from growth to decay, which is fixed at 35 days (5 weeks post first vaccination) for both human and mice based on literature indicating peak timing of 28–42 days in mice^48,49^ and ~ 5 weeks in humans^50,51^. To ensure the identifiability and comparability of the kinetic parameters ${k}_{1}$ and ${k}_{2}$ across different doses and species, we maintained a constant $\tau$ to prevent parameter correlation during estimation. The robustness of this structural assumption was validated by comparing the primary model against an alternative model where $\tau$ was estimated with a random effect; model selection was based on the Akaike Information Criterion (AIC) and Bayesian Information Criterion (BIC).

The model was independently fitted to the mice and human data using nonlinear mixed effects modelling in Monolix2024R1. Log-normal distribution was assumed for the random effect of the parameters ${A}_{0}, {k}_{1},{k}_{2},$ while $C$ was modeled with a logit-normal distribution bounded between 0 and 100. For the mice data, dose was implemented as a categorical covariate on parameters ${k}_{1}$ and ${k}_{2}$. This approach avoids imposing an assumed linear or continuous relationship between numeric dose and parameter values, thereby reducing potential bias from an arbitrary functional form. If we set dose group 0.05 μg as the reference group, the resulting equation for parameter $k$ is:

$$log(k)= log\left({k}_{pop}\right)+\sum_{d\in \left\{\mathrm{0.1,0.2,0.5,1}\right\}}{\beta}_{k,d}*1\left[Vaccine=d \mu g\right]+ \eta k$$

where $k$ can be ${k}_{1}$ or ${k}_{2}$; ${\beta}_{k,d}$ represents the effect of dose group $d$ on parameter $k$, $1\left[Vaccine=d \mu g\right]$ is the indicator function taking value 1 if the condition is true and 0 otherwise; and $\eta k$ is the individual-level random effect.

B) Process 2. Assessment of the association between the model parameters and dose in mice data. We fitted various linear and non-linear models (logarithm, exponential, and power-law) to model the association between the point estimate of the model parameters (${k}_{1}$ and ${k}_{2}$) and dose. We selected the best model with the lowest AIC and the highest ${R}^{2}$, which was utilized in the further process. The candidate equations tested were:

- Linear: $$p\left(D\right)=a+b*D$$
- Exponential: $$p\left(D\right)=a*exp(-b*D)$$
- Logarithm: $$p\left(D\right)=ln(a*D-b)$$
- Power-law: $$p\left(D\right)=a*{D}^{-b}$$

C) Process 3. Extrapolation of antibody response to vaccination in human under different doses using empirical dose translation.

The translation factor $s$ is used to translate mice dose (${dose}_{m}$) to human dose (${dose}_{h}$): ${dose}_{h}=s*{dose}_{m}$. Assuming the parameter $p$ for mice is modelled as ${p}_{m}({dose}_{m})=f({dose}_{m})$ ($f$ is determined in Process 2), and the parameter $p$ for human is estimated as ${p}_{h}\left({dose}_{h}^{*}\right)={p}_{h}^{*}$ (estimated in Process 1), where ${dose}_{h}^{*}$ is the dose administrated in human trial, the parameter for human under different doses ($dose_{h}$) is estimated as follow: $p_{h} \left( { dose_{h} } \right) = p_{h}^{*} \left( {1 + \frac{{p_{m} \left( {dose_{h} /s} \right)}}{{p_{m} \left( {dose_{h}^{*} /s} \right)}}} \right)$. We note that the antibody response under the same vaccination doses after translation (i.e., e.g., , 0.05 μg for mice corresponds to 5 μg for human) could be different (i.e., ${p}_{m}({dose}_{m})\ne {p}_{h}({dose}_{h})$), which represents the difference between the species. We assumed a translation factor of 100^39^, and the human 30 μg dose (0.3 μg in mice) as baseline. While this factor is used to facilitate the empirical translation, it is not intended as a definitive biological conversion; our sensitivity analysis confirms that the qualitative conclusions remain robust across a range of translation factors.

### Statistical analysis

Statistical analysis was performed using GraphPad Prism 9. Shapiro–Wilk test was applied to normalized log-transformed values to assess the normality. One-way ANOVA was then performed on normalized log-transformed values, followed by post-hoc tests. Tukey’s multiple comparisons test was used to correct for multiple comparisons. All tests were two-tailed, and *p* < 0.05 was considered statistically significant. To formally assess the equivalence of kinetic parameters between species, we performed a Two One-Sided Test (TOST) using the TOSTER package in R. Equivalence margins were defined as ± 2 standard deviations of the difference between the mouse and human parameter means. This margin was selected to provide a conservative threshold for equivalence that accounts for the variance in longitudinal antibody titters.

## Supplementary Information

Supplementary Information 1.

Supplementary Information 2.

## Data Availability

All data and its Supplementary files in the article are available from the corresponding author upon reasonable request.
