# Supplementary material for: Optimizing mouse models for mRNA vaccines: addressing dose translation challenges
Source: Sci Rep. 2026 May 5;16:20692. doi: 10.1038/s41598-026-47820-z (PMC13333994; doi:10.1038/s41598-026-47820-z)
Supplement: Supplementary file 1 — Supplementary Information 1. [file 41598_2026_47820_MOESM1_ESM.pptx]

## Slide 1
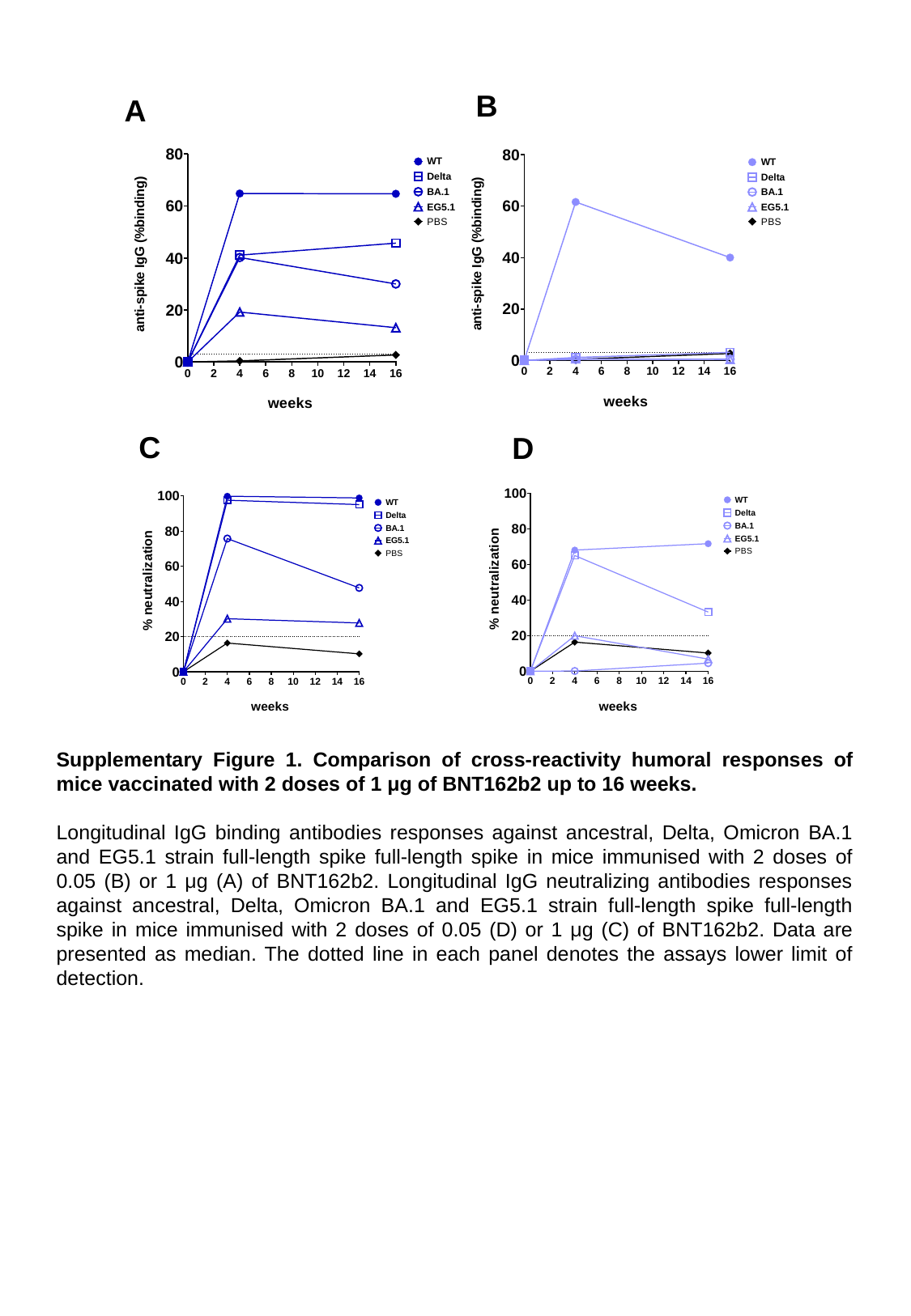

B
A
C
D
Supplementary Figure 1. Comparison of cross-reactivity humoral responses of mice vaccinated with 2 doses of 1 μg of BNT162b2 up to 16 weeks.
Longitudinal IgG binding antibodies responses against ancestral, Delta, Omicron BA.1 and EG5.1 strain full-length spike full-length spike in mice immunised with 2 doses of 0.05 (B) or 1 μg (A) of BNT162b2. Longitudinal IgG neutralizing antibodies responses against ancestral, Delta, Omicron BA.1 and EG5.1 strain full-length spike full-length spike in mice immunised with 2 doses of 0.05 (D) or 1 μg (C) of BNT162b2. Data are presented as median. The dotted line in each panel denotes the assays lower limit of detection.

## Slide 2
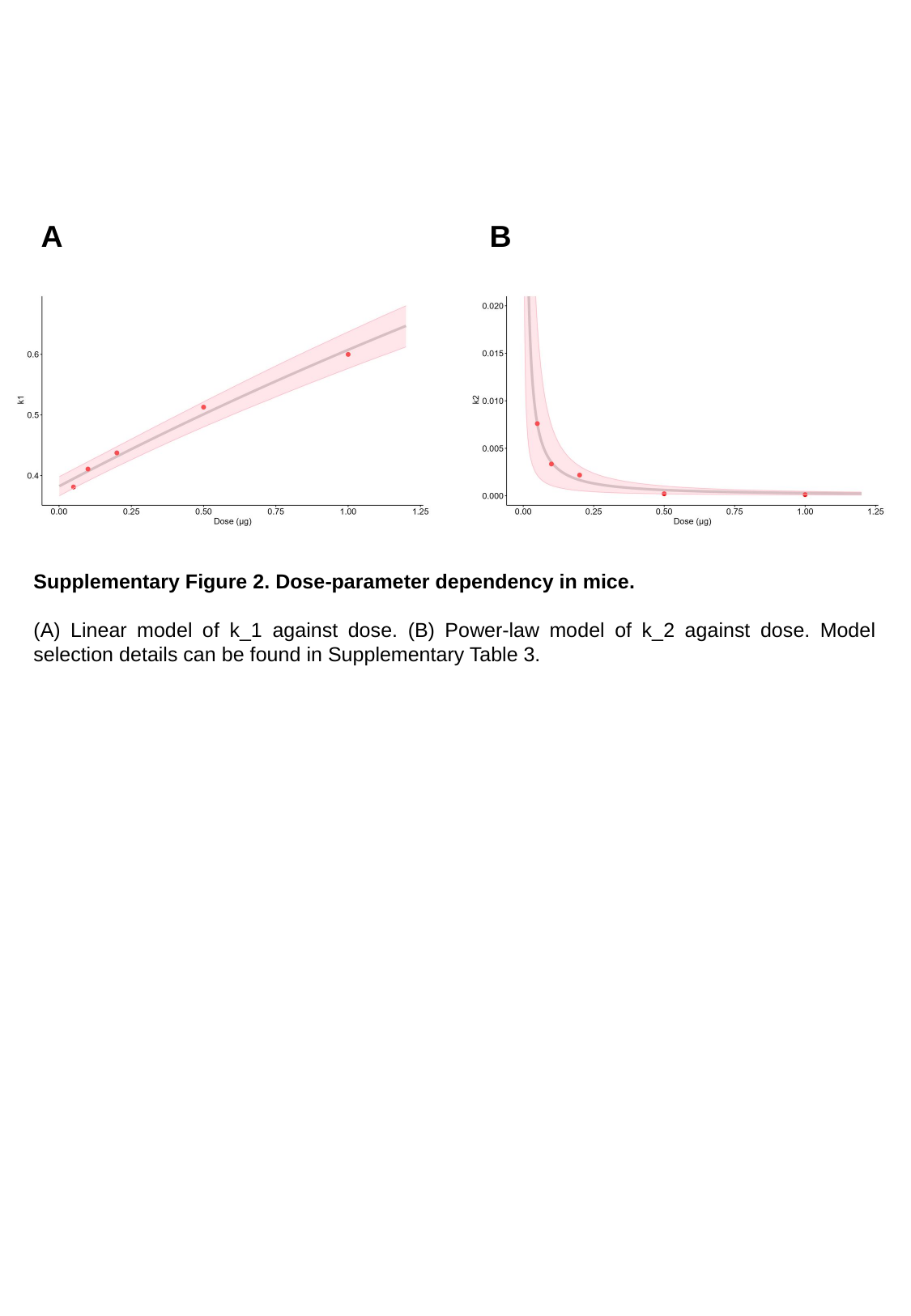

A
B
Supplementary Figure 2. Dose-parameter dependency in mice.
(A) Linear model of k_1 against dose. (B) Power-law model of k_2 against dose. Model selection details can be found in Supplementary Table 3.

## Slide 3
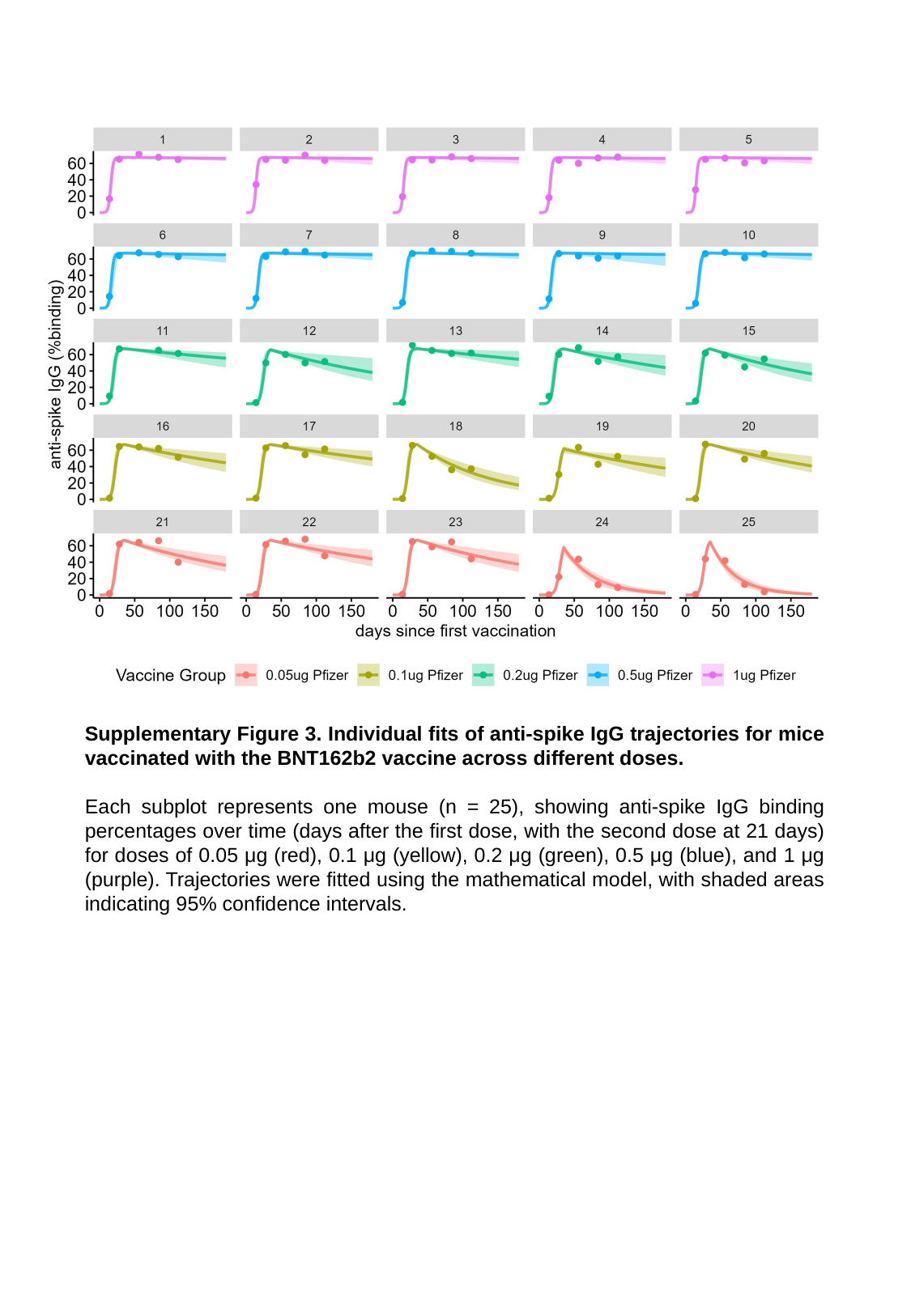

Supplementary Figure 3. Individual fits of anti-spike IgG trajectories for mice vaccinated with the BNT162b2 vaccine across different doses.
Each subplot represents one mouse (n = 25), showing anti-spike IgG binding percentages over time (days after the first dose, with the second dose at 21 days) for doses of 0.05 μg (red), 0.1 μg (yellow), 0.2 μg (green), 0.5 μg (blue), and 1 μg (purple). Trajectories were fitted using the mathematical model, with shaded areas indicating 95% confidence intervals.

## Slide 4
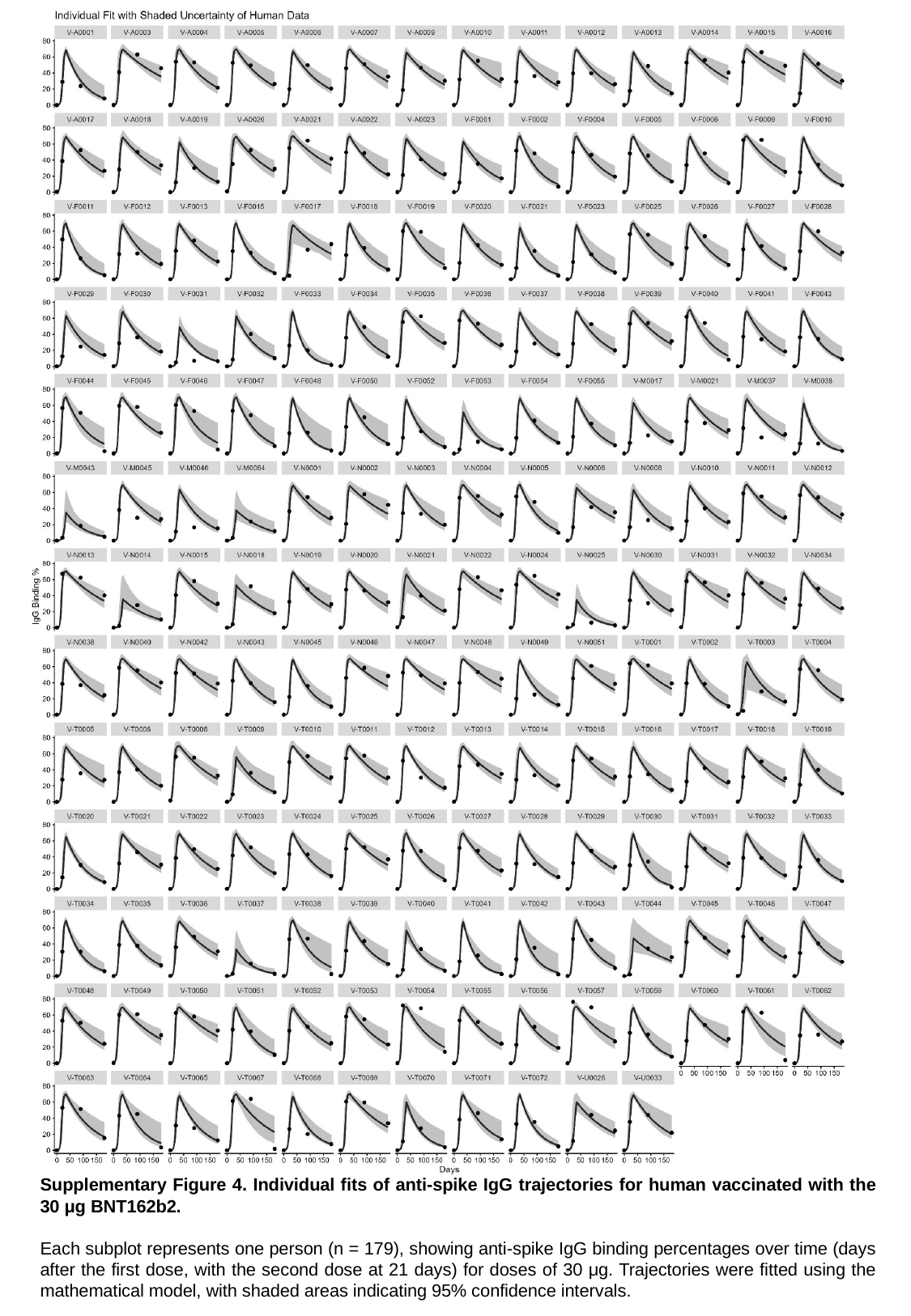

Supplementary Figure 4. Individual fits of anti-spike IgG trajectories for human vaccinated with the 30 μg BNT162b2.
Each subplot represents one person (n = 179), showing anti-spike IgG binding percentages over time (days after the first dose, with the second dose at 21 days) for doses of 30 μg. Trajectories were fitted using the mathematical model, with shaded areas indicating 95% confidence intervals.

## Slide 5
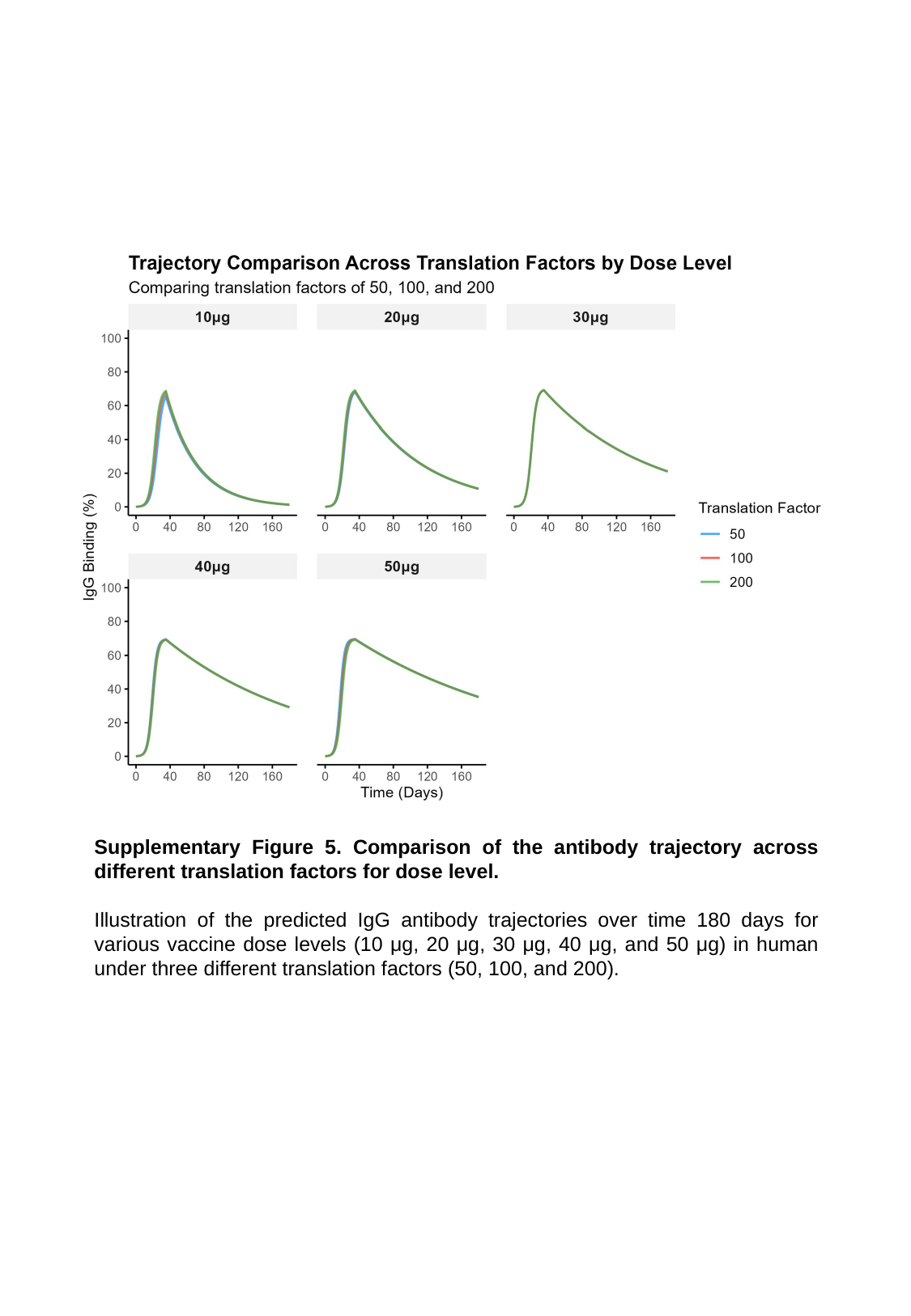

Supplementary Figure 5. Comparison of the antibody trajectory across different translation factors for dose level.
Illustration of the predicted IgG antibody trajectories over time 180 days for various vaccine dose levels (10 μg, 20 μg, 30 μg, 40 μg, and 50 μg) in human under three different translation factors (50, 100, and 200).

## Slide 6
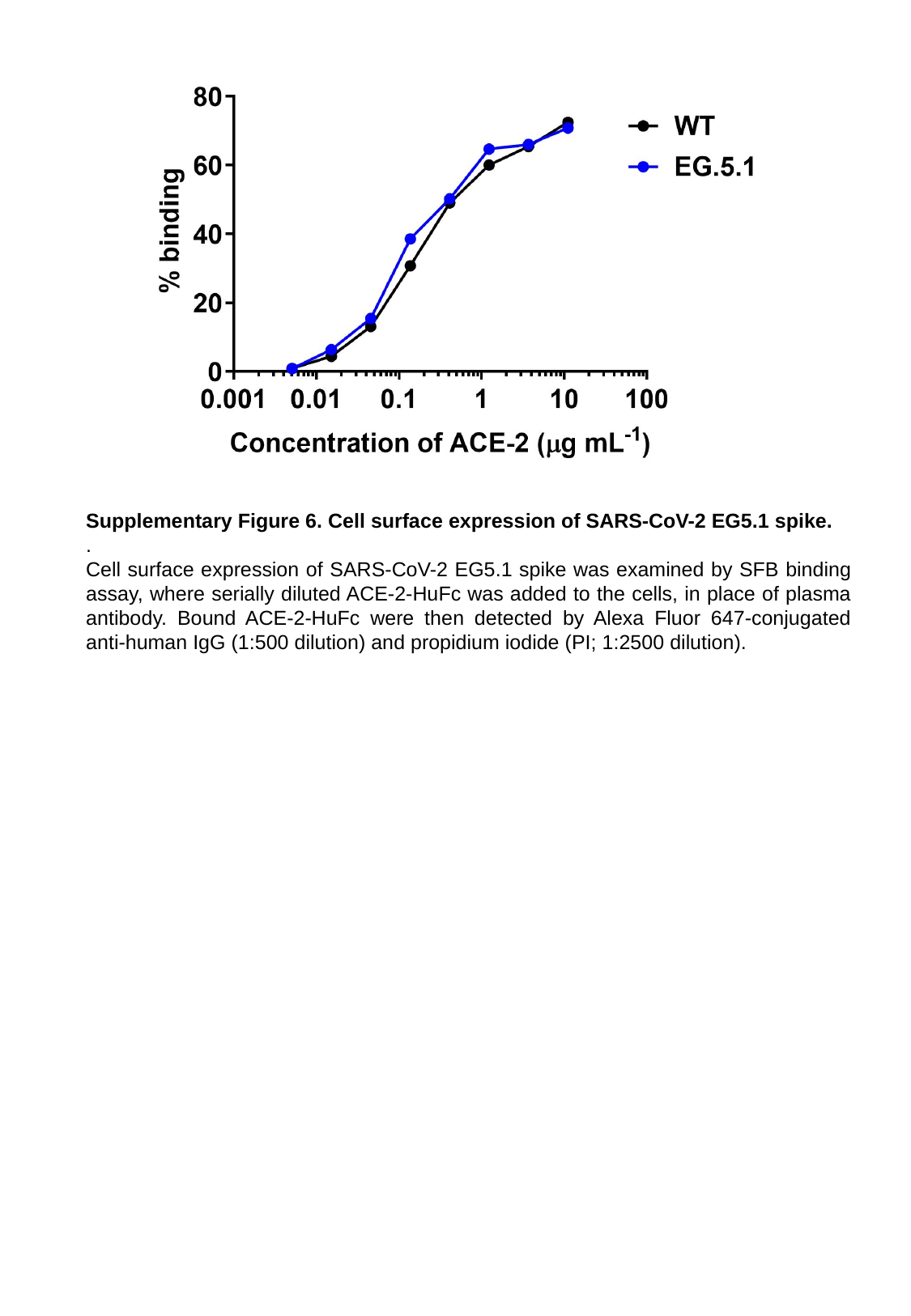

Supplementary Figure 6. Cell surface expression of SARS-CoV-2 EG5.1 spike.
.
Cell surface expression of SARS-CoV-2 EG5.1 spike was examined by SFB binding assay, where serially diluted ACE-2-HuFc was added to the cells, in place of plasma antibody. Bound ACE-2-HuFc were then detected by Alexa Fluor 647-conjugated anti-human IgG (1:500 dilution) and propidium iodide (PI; 1:2500 dilution).

## Slide 7
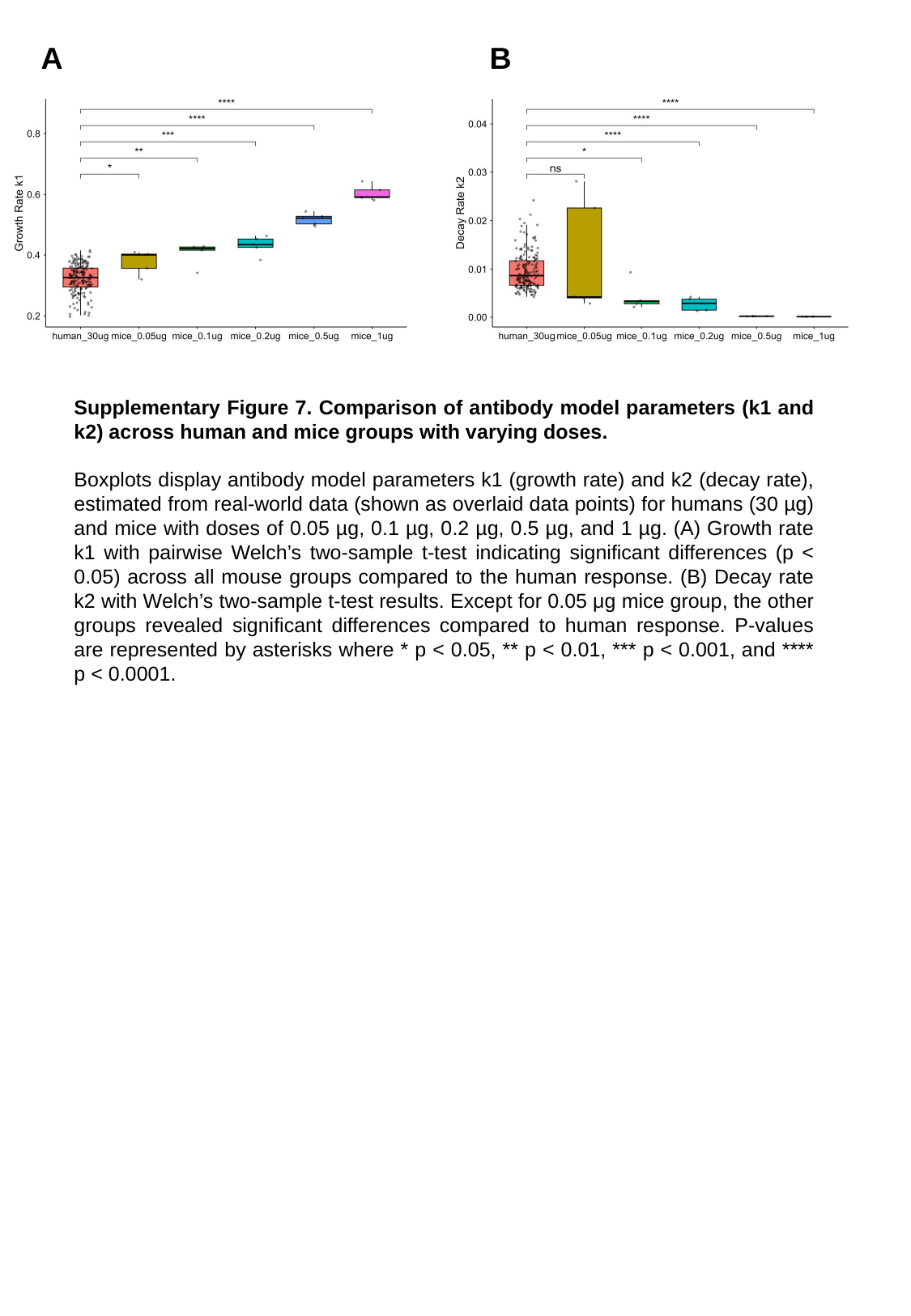

A
B
Supplementary Figure 7. Comparison of antibody model parameters (k1 and k2) across human and mice groups with varying doses.
Boxplots display antibody model parameters k1 (growth rate) and k2 (decay rate), estimated from real-world data (shown as overlaid data points) for humans (30 µg) and mice with doses of 0.05 µg, 0.1 µg, 0.2 µg, 0.5 µg, and 1 µg. (A) Growth rate k1 with pairwise Welch’s two-sample t-test indicating significant differences (p < 0.05) across all mouse groups compared to the human response. (B) Decay rate k2 with Welch’s two-sample t-test results. Except for 0.05 μg mice group, the other groups revealed significant differences compared to human response. P-values are represented by asterisks where * p < 0.05, ** p < 0.01, *** p < 0.001, and **** p < 0.0001.
