# Supplementary material for: Optimizing mouse models for mRNA vaccines: addressing dose translation challenges
Source: Sci Rep. 2026 May 5;16:20692. doi: 10.1038/s41598-026-47820-z (PMC13333994; doi:10.1038/s41598-026-47820-z)
Supplement: Supplementary file 2 — Supplementary Information 2. [file 41598_2026_47820_MOESM2_ESM.docx]

**Supplementary Table 1: Population parameters of sigmoid-exponential model for mice data.**

| Parameter (unit) | Symbol | Population Mean | Standard error | Standard deviation of random effect | Distribution | Source |
| --- | --- | --- | --- | --- | --- | --- |
| Antibody growth rate (${day}^{-1}$) | $k1$ | 0.38 | 0.027 | 0.089 | Log-normal | estimated |
| Antibody decay rate (${day}^{-1}$) | $k2$ | 0.0076 | 0.003 | 0.765 | Log-normal | estimated |
| Initial antibody level (%) | $A_{0}$ | 0.007 | 0.0086 | 0.39 | Log-normal | estimated |
| Binding capacity (%) | $C$ | 67.11 | 0.79 | 0.0078 | Logit-normal | estimated |
| Onset of transition from growth to decay ($day$) | $\tau$ | 35 | --- | --- | --- | fixed |
| Log-scale change in k1 ​ for 0.1 μg vs. 0.05 μg | $\beta_{k1-0.1\mu g}$ | 0.075 | 0.071 | --- | --- | estimated |
| Log-scale change in k1 ​ for 0.2 μg vs. 0.05 μg | $\beta_{k1-0.2\mu g}$ | 0.14 | 0.077 | --- | --- | estimated |
| Log-scale change in k1 ​ for 0.5 μg vs. 0.05 μg | $\beta_{k1-0.5\mu g}$ | 0.30 | 0.10 | --- | --- | estimated |
| Log-scale change in k1 ​ for 1 μg vs. 0.05 μg | $\beta_{k1-1\mu g}$ | 0.45 | 0.082 | --- | --- | estimated |
| Log-scale change in k2​ for 0.1 μg vs. 0.05 μg | $\beta_{k2-0.1\mu g}$ | -0.82 | 0.53 | --- | --- | estimated |
| Log-scale change in k2 ​ for 0.2 μg vs. 0.05 μg | $\beta_{k2-0.2\mu g}$ | -1.25 | 0.54 | --- | --- | estimated |
| Log-scale change in k2 ​ for 0.5 μg vs. 0.05 μg | $\beta_{k2-0.5\mu g}$ | -3.56 | 1.29 | --- | --- | estimated |
| Log-scale change in k2 ​ for 1 μg vs. 0.05 μg | $\beta_{k2-1\mu g}$ | -4.092 | 1.27 | --- | --- | estimated |
| Constant error parameter | $a$ | 4.44 | 0.36 | --- | --- | estimated |

**Supplementary Table 2: Population parameters of sigmoid-exponential model for human data.**

| Parameter (unit) | Symbol | Population Mean | Standard error | Standard deviation of random effect | Distribution | Source |
| --- | --- | --- | --- | --- | --- | --- |
| Antibody growth rate (${day}^{-1}$) | $k1$ | 0.33 | 0.006 | 0.19 | Log-normal | estimated |
| Antibody decay rate (${day}^{-1}$) | $k2$ | 0.0082 | 0.003 | 0.42 | Log-normal | estimated |
| Initial antibody level (%) | $A_{0}$ | 0.078 | 0.0086 | 0.82 | Log-normal | estimated |
| Binding capacity (%) | $C$ | 69.68 | 0.79 | 0.034 | Logit-normal | estimated |
| Onset of transition from growth to decay ($day$) | $\tau$ | 35 | --- | --- | --- | fixed |
| Proportional error parameter | $b$ | 0.27 | 0.012 | --- | --- | estimated |

**Supplementary Table 3: Comparison of model fit for fixed vs. estimated transition time** $\boldsymbol{\tau}$

| **Model** | **Data** | $\boldsymbol{\tau}$ **(days)** | **AIC** | **BIC** |
| --- | --- | --- | --- | --- |
| Primary Model | Mice | Fixed (35) | 819 | 832 |
| Alternative Model | Mice | Estimated | 818 | 835 |
| Primary Model | Human | Fixed (35) | 3853 | 3878 |
| Alternative Model | Human | Estimated | 3861 | 3890 |

**Supplementary Table 4: Comparing various dose-parameter dependency models based on AIC and R² values to determine the best-fit model**

The models with lowest AIC and highest R^2^ are suggested by underlines.

| **Parameters** | **Model** | **AIC** | $\boldsymbol{R}^{\mathbf{2}}$ |
| --- | --- | --- | --- |
| $k_{1}$ - Dose | linear | -24.73 | 0.980 |
| $k_{1}$ - Dose | exponential | -21.77 | 0.964 |
| $k_{1}$ - Dose | power-law | -22.93 | 0.971 |
| $k_{1}$ - Dose | logarithm | **-26.65** | **0.986** |
| $k_{2}$ - Dose | linear | -42.90 | 0.558 |
| $k_{2}$ - Dose | exponential | -54.96 | 0.960 |
| $k_{2}$ - Dose | power-law | **-60.50** | **0.987** |
| $k_{2}$ - Dose | logarithm | -42.91 | 0.559 |

**Supplementary Table 5: Estimated Coefficients for Dose-Parameter Relationships**

| **Parameter** | **Equation** | **Coefficient a** | **Coefficient b** |
| --- | --- | --- | --- |
| Growth rate $k_{1}$ | $ln(a*D-b)$ | 0.370 | -1.466 |
| Decay rate $k_{2}$ | $a*D^{-b}$ | 2.86×10-4 | 1.094 |
